# Annual acknowledgement of manuscript reviewers

**DOI:** 10.1186/s12954-015-0036-3

**Published:** 2015-03-20

**Authors:** Nick Crofts, Euan Lawson

**Affiliations:** Co-Editors-in-Chief, Harm Reduction Journal, ᅟ, ᅟ

**Keywords:** ■■■

## Abstract

The editors of *Harm Reduction Journal* would like to thank all our reviewers who have contributed to the journal in Volume 11 (2014).

**Noorizan Abd.Aziz**

Malaysia

**Campbell Aitken**

Australia

**Scott Anderson**

Canada

**Shervin Assari**

United States of America

**Thomas Babor**

United States of America

**Jo Barnes**

New Zealand

**Monica Barratt**

Australia

**Barbara Barrett**

United Kingdom

**Angela Bazzi**

United States of America

**Tolesa Bekele**

Ethiopia

**Leo Beletsky**

United States of America

**David Bewley-Taylor**

United Kingdom

**Ricky Bluthenthal**

United States of America

**Adam Bourne**

United Kingdom

**Sarah Byford**

United Kingdom

**Agnes Cadet-Taïrou**

France

**Jacqui Cameron**

Australia

**Ross Coomber**

United Kingdom

**Charles Cornford**

United Kingdom

**Karen Corsi**

United States of America

**Ladislav Csemy**

Czech Republic

**Daryle Deering**

New Zealand

**Mitch Earleywine**

United States of America

**Karl Fagerstrom**

Sweden

**Niamh Fingleton**

United Kingdom

**Gabriele Fischer**

Austria

**Chris Ford**

United Kingdom

**Samuel Friedman**

United States of America

**Tommi Gaines**

United States of America

**Elizabeth Golub**

United States of America

**Lauretta Grau**

United States of America

**Carsten Grimm**

United Kingdom

**Jean-Paul Grund**

Netherlands

**Robert Haemmig**

Switzerland

**Magdalena Harris**

United Kingdom

**Dagmar Hedrich**

Portugal

**Peter Higgs**

Australia

**Richard Holland**

United Kingdom

**Neil Hunt**

United Kingdom

**Devon Indig**

Australia

**M. Mofizul Islam**

Australia

**Mikael Jansson**

Canada

**Pratap Jena**

India

**Ehsan Jozaghi**

Canada

**Angela Kaida**

Canada

**Irma Kirtadze**

Georgia

**Mark Kleiman**

United States of America

**John-Peter Kools**

Netherlands

**M. Suresh Kumar**

India

**Briony Larance**

Australia

**Carl Latkin**

United States of America

**Charlie Lloyd**

United Kingdom

**Kirsten Marchand**

Canada

**Catriona Matheson**

United Kingdom

**Andrew McAuley**

United Kingdom

**Sheryl McCurdy**

United States of America

**Sanjay Mehendale**

India

**Ingo Michels**

Germany

**M-J Milloy**

Canada

**Caroline Mitchell**

United Kingdom

**Felix Naughton**

United Kingdom

**Zbynek Oktabec**

Czech Republic

**Sonja Pasche**

South Africa

**Amber Pearson**

New Zealand

**Carl Phillips**

Canada

**Barbara Pieper**

United States of America

**Polly Radcliffe**

United Kingdom

**Emran Razaghi**

Iran

**Umar Saeed**

Pakistan

**Riza Sarasvita**

Indonesia

**Mary Philip Sebastian**

India

**Mukta Sharma**

Thailand

**Laura Sheard**

United Kingdom

**Janie Sheridan**

New Zealand

**Susan Sherman**

United States of America

**Derek Smith**

Australia

**Magnus Stenbeck**

Sweden

**Carol Strike**

Canada

**Bengt Svensson**

Sweden

**Alfred Uhl**

Austria

**Marie Claire Van Hout**

Ireland

**Tord Finne Vedoy**

Norway

**Andrea Wirtz**

United States of America

**Alex Wodak**

Australia

